# Novel Polyprenylated Phloroglucinols from *Hypericum sampsonii*

**DOI:** 10.3390/molecules191219836

**Published:** 2014-11-28

**Authors:** Jih-Jung Chen, Hong-Jhang Chen, Yun-Lian Lin

**Affiliations:** 1 Department of Pharmacy, Tajen University, Pingtung 907, Taiwan; E-Mail: jjchen@tajen.edu.tw; 2 Graduate Institute of Pharmaceutical Technology, Tajen University, Pingtung 907, Taiwan; 3 National Research Institute of Chinese Medicine, Taipei 112, Taiwan; E-Mail: f92641017@ntu.edu.tw; 4 School of Pharmacy, National Taiwan University, Taipei 100, Taiwan

**Keywords:** *Hypericum sampsonii*, Guttiferae, polyprenylated phloroglucinol

## Abstract

*Hypericum sampsonii* Hance (Clusiaceae) is a folk medicine used in Taiwan to treat blood stasis, relieve swelling, and as an anti-hepatitis drug. Two new polyprenylated phloroglucinol derivatives, hypersampsone R (**1**) and hypersampsone S (**2**), and a known prenylated benzophenone, hyperibone K (**3**) were isolated from the aerial parts of *H. sampsonii*. Their structures were determined by extensive 1D and 2D NMR, and MS spectral analyses.

## 1. Introduction

**Figure 1:**
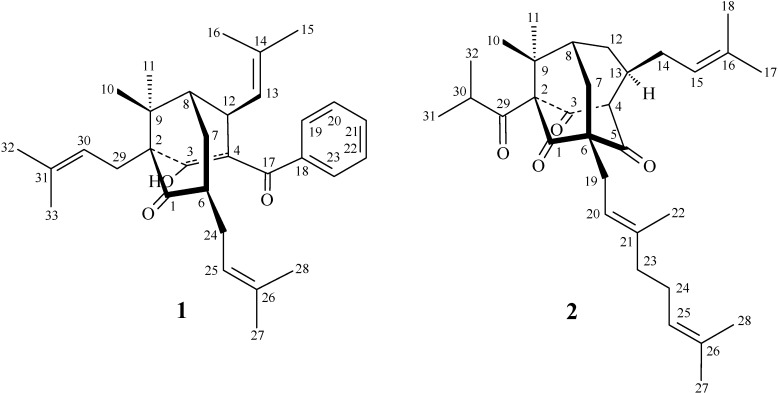
The chemical structures of new compounds **1** and **2** isolated from *H. sampsonii*.

**Figure 2:**
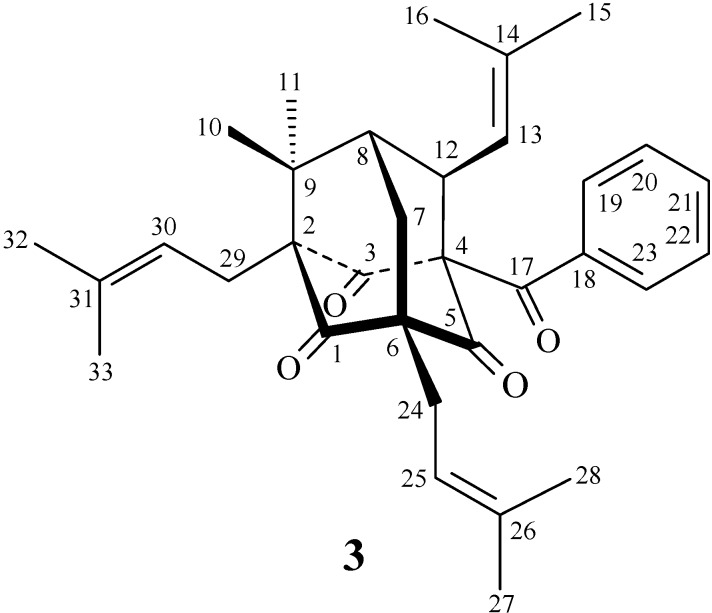
The chemical structure of known compound **3** isolated from *H. sampsonii*.

*Hypericum sampsonii* Hance (Guttiferae) is a folk herbal medicine used in Taiwan for treating blood stasis, to relieve swelling, and as an anti-hepatitic drug [1]. Due to not only the biological activities, but also the structural diversity, the chemical constituents of *Hypericum* species have attracted much attention, and different kinds of compounds such as xanthones [2,3,4,5], benzophenones [5,6], bisanthraquinones [6,7], and polyprenylated phloroglucinols [8,9,10,11,12,13,14,15,16] have been isolated. A continuing chemical investigation on the secondary metabolites of this plant resulted in the isolation of a new ring-opened polyprenylated benzophenone, hypersampsone R (**1**) and a new polyprenylated phloroglucinol, hypersampsone S (**2**) (Figure 1), as well as a known polyprenylated benzophenone, hyperibone K (**3**) (Figure 2). This paper describes the structural elucidation of compounds **1** and **2**.

## 2. Results and Discussion

The ethanol extract of the air-dried aerial parts of *H. sampsonii* was successively partitioned with ethyl acetate (EtOAc) and *n*-butanol (*n*-BuOH) to give EtOAc, *n*-BuOH and H_2_O fractions. The EtOAc soluble partition enriched in polyprenylated phloroglucinols was subjected to silica gel and RP-18 column chromatography in combination with preparative silica-gel HPLC to yield two new compounds **1** and **2**, together with a known compound **3**.

**Figure 3:**
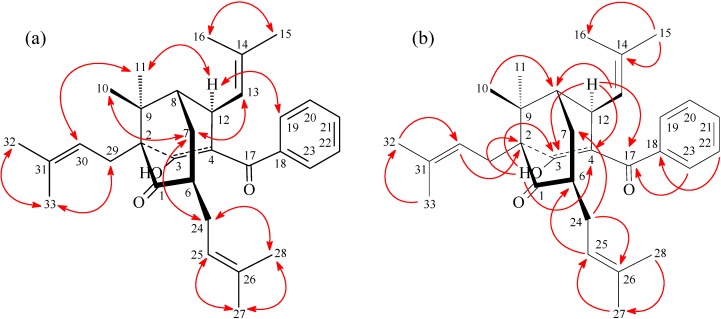
Key NOESY (**a**) and HMBC (**b**) correlations of **1**.

Hypersamsone R (**1**) was isolated as an optically active (${\left[\alpha \right]}_{D}^{25}$ = +160°), colorless amorphous powder. The molecular formula was established as C_32_H_42_O_3_ on the basis of HR-EI-MS (found *m/z* 474.3128, calcd. for C_32_H_42_O_3_ 474.3134) with twelve indices of hydrogen deficiency (IHD). The IR spectrum displayed absorptions of hydroxyl (3443 cm^−1^) and carbonyl groups (1709 and 1677 cm^−1^). The ^1^H-NMR spectrum of **1** showed three olefinic protons [δ_H_ 4.63 (1H, d, *J* = 7.8 Hz), 4.98 (1H, t, *J* = 6.5 Hz), and 5.15 (br s)], eight methyls [δ_H_ 0.92, 1.18, 1.24, 1.26, 1.54, 1.56, 1.65, and 1.66 (each 3H, s)], a benzoyl [δ_H_ 7.36 (3H, m) and 7.39 (2H, m)], and a conjugated hydroxyl group [δ_H_ 16.25 (s)]. The ^13^C-NMR, DEPT and HMQC spectra indicated the presence of 32 carbons, including two carbonyls (δ_C_ 195.2 and 209.4), eight quaternary carbons with one conjugated oxygenated quaternary carbon (δ_C_ 185.5), five fully substituted aromatic and olefinic quarternary carbons (δ_C_ 111.4, 130.3, 132.7, 133.2, and 139.3) and two quaternary carbons (δ_C_ 42.4 and 65.4), eight double-bond methine carbons [δ_C_ 121.3, 122.9, 125.7, 126.7, 126.7, 128.0, 128.0 and 130.1], three methine carbons [δ_C_ 35.4, 45.3, 45.5], three methylene carbons [δ_C_ 26.3, 28.3, 29.9], and eight methyl carbons [δ_C_ 17.3, 17.9, 17.9, 25.1, 25.1, 25.7, 26.0, 26.0]. The ^1^H-^1^H COSY indicated the correlations of H-12 (δ_H_ 4.00) and H-13 (δ_H_ 4.63) and H-8 (δ_H_ 1.50); H-6 (δ_H_ 2.50) and H-7 (δ_H_ 1.44, 2.06) and H-24 (δ_H_ 2.32). Comparison of the ^1^H- and ^13^C-NMR data (Table 1) of **1** with those of hyperibone K (**3**) [17] suggested that their structures were closely related, except that the C3,C4-double bond, 3-hydroxy group, and the lack of carbonyl group between C-4 and C-6 of **1** replaced the C3,C4-single bond and 3-oxo group of hyperibone K (**3**) [17]. This was supported by HMBC correlations (Figure 3) between OH-3 (δ_H_ 16.25) and C-2 (δ_C_ 65.4), C-3 (δ_C_ 185.5), and C-4 (δ_C_ 111.4); between H-12 (δ_H_ 4.00) and C-3 (δ_C_ 185.5), C-4 (δ_C_ 111.4), C-7 (δ_C_ 29.9), C-8 (δ_C_ 45.3), C-13 (δ_C_ 125.7) and C-14 (δ_C_ 132.7). The NOESY cross-peaks (Figure 3) between H-7/Me(10), H-7/H-13, H-7/H-24, H_α_-12/H-19, H_α_-12/Me(11), and H-30/Me(11) suggested that H-12, Me(11), 2-isoprenyl, and the 4-benzoyl groups are α-oriented, and 6-isoprenyl, Me(10), and C-12 2-methylprop-1-enyl groups are β-oriented. The full assignment of ^1^H- and ^13^C-NMR resonances was supported by ^1^H-^1^H COSY, DEPT, HMQC, NOESY (Figure 3), and HMBC (Figure 3) spectral analyses. According to the above data, hypersamsone R was identified as structure **1**.

**Table 1:** ^1^H- and ^13^C-NMR data of **1** and **2**.

Position	δ_H_			
	1 ^a^		2 ^a^	
	^1^H	^13^C	^1^H	^13^C
1		209.4 s		202.2 s
2		65.4 s		88.8 s
3		185.5 s		205.4 s
4		111.4 s	2.97 m	50.0 d
5				204.7 s
6	2.50 m	45.5 d		69.2 s
7	1.44 m	29.9 t	1.87 d (10.5)	36.2 t
	2.06 m		2.50 m	
8	1.50 m	45.3 d	1.75 m	41.4 d
9		42.4 s		50.0 s
10	1.26 s	26.0 q	1.20 s	24.8 q
11	0.92 s	25.1 q	1.18 s	24.2 q
12	4.00 dd (7.8, 7.0)	35.4 d	1.84 d (7.8)	35.4 d
			2.74 m	
13	4.63 d (7.8)	125.7 d	2.81 m	58.1 d
14		132.7 s	2.06 m	31.1 t
			2.30 m	
15	1.24 s	25.1 q	4.97 t (6.5)	119.9 d
16	1.18 s	17.3 q		135.8 s
17		195.2 s	1.67 s	25.8 q
18		139.3 s	1.56 s	17.9 q
19	7.36 m	128.0 d	2.50 m	32.3 t
			2.66 dd (11.5, 7.0)	
20	7.39 m	126.7 d	4.99 t (7.0)	119.3 d
21	7.36 m	130.1 d		139.6 s
22	7.39 m	126.7 d	1.61 s	16.4 q
23	7.36 m	128.0 d	2.01 m	40.1 t
24	1.88 m	28.3 t	2.03 m	26.6 t
	2.32 m			
25	4.98 t (6.5)	121.3 d	5.00 t (6.5)	124.1 d
26		133.2 s		131.5 s
27	1.65 s	26.0 q	1.64 s	25.7 q
28	1.54 s	17.9 q	1.56 s	17.6 q
29	2.57 dd (13.0, 7.0)	26.3 t		211.5 s
	2.89 br d (13.0)			
30	5.15 br s	122.9 d	2.45 hepta (7.0)	42.8 d
31		130.3 s	1.19 d (7.0)	21.0 q
32	1.66 s	25.7 q	1.21 d (7.0)	21.0 q
33	1.56 s	17.9 q		
OH	16.25 s			

^a^ Recorded in CDCl_3_ at 500 MHz (^1^H) and 125 MHz (^13^C). Values in ppm (δ). *J* (in Hz) in parentheses.

**Figure 4:**
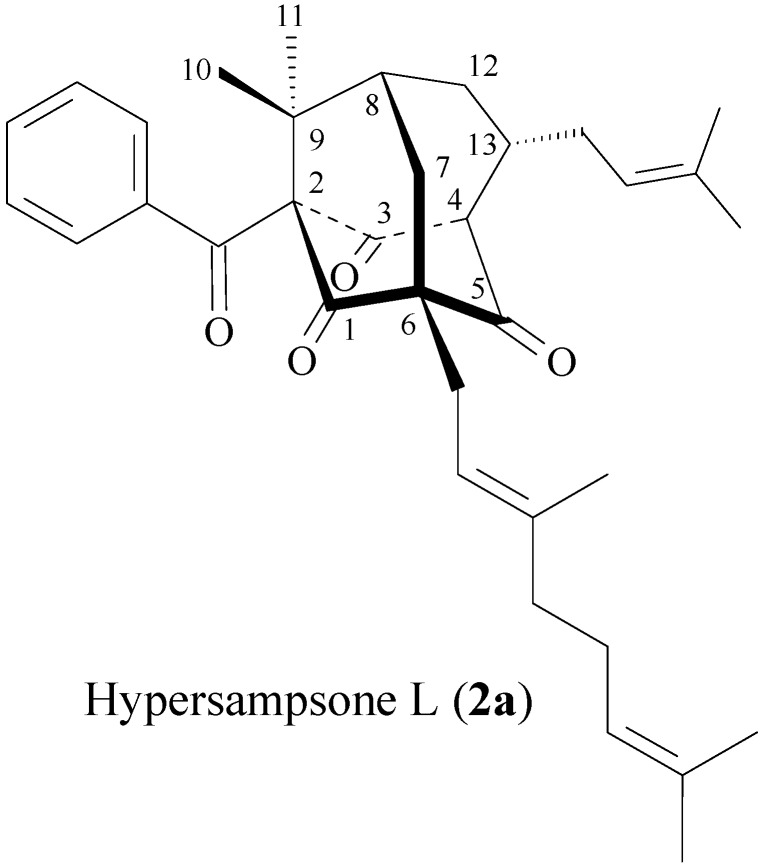
The chemical structure of hypersampsone L (**2a**).

**Figure 5:**
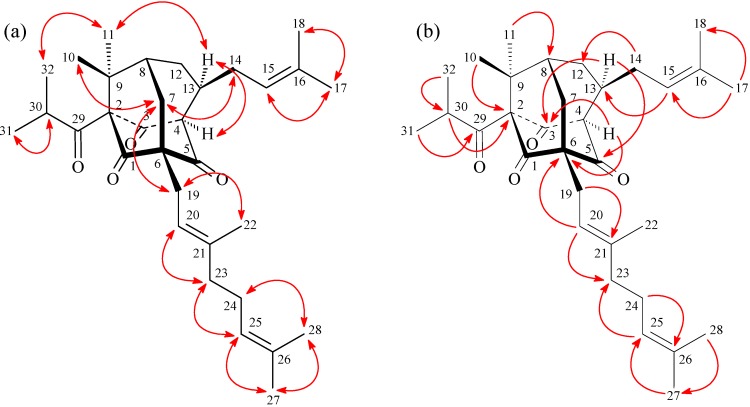
Key NOESY (**a**) and HMBC (**b**) correlations of **2**.

Hypersamsone S (2) was obtained as a colorless amorphous powder. The molecular formula was determined to be C_3__2_H_46_O_4_ on the basis of HR-EI-MS (found *m/z* 494.3396, calcd. for C_3__2_H_46_O_4_ 494.3394) with nine IHD. The ^13^C-NMR, DEPT and HMQC spectra indicated ten quaternary carbons [including four carbonyl (δ_C_ 202.2, 204.7, 205.4, and 211.5), three olefinic quaternary (δ_C_ 131.5, 135.8, and 139.6), and three other quaternary carbons (δ_C_ 50.0, 69.2, and 88.8)], seven tertiary carbons [including three olefinic (δ_C_ 119.3, 119.9, and 124.1) and four other tertiary carbons (δ_C_ 41.4, 42.8, 50.0, and 58.1)], and six methylene carbons (δ_C_ 26.6, 31.1, 32.3, 35.4, 36.2, and 40.1), and nine methyl carbons (δ_C_ 16.4, 17.6, 17.9, 21.0, 21.0, 24.2, 24.8, 25.7, and 25.8). Comparison of the ^1^H- and ^13^C-NMR data (Table 1) of 2 with those of hypersampsone L (2a) [18] suggested that their structures were closely related, except that 2-isobutyryl and 13β-isoprenyl groups of 2 replaced 2-benzoyl and 13α-isoprenyl groups of hypersampsone L (**2a**, Figure 4) [18]. This was supported by HMBC correlations (Figure 5) between H-15 (δ_H_ 4.97) and C-13 (δ_C_ 58.1); between H-20 (δ_H_ 4.99) and C-6 (δ_C_ 69.2); and between H-30 (δ_H_ 2.45) and C-2 (δ_C_ 88.8). The NOESY cross-peaks (Figure 5) between H_α_-4/ H_α_-13, H_α_-13/Me(11), H-32/Me(11), H-7/Me(10), H-7/H-14, H-7/H-19, suggested that H_α_-4, H_α_-13, Me(11), and the 2-isobutyryl groups are α-oriented, and 6-geranyl, Me(10), and 13-isoprenyl groups are β-oriented. On the basis of the evidence above, the structure of hypersamsone S was elucidated as 2, which was further substantiated through 2D-experiments, including HMQC, ^1^H-^1^H COSY, HMBC (Figure 5), and NOESY (Figure 5) spectra.

The known isolate, hyperibone K (**3**) was readily identified by a comparison of physical and spectroscopic data (IR, ^1^H-NMR, [α]_d_, and MS) with the literature values [17].

## 3. Experimental Section

### 3.1. General Experimental Procedures

Optical rotations were measured using a Jasco P-2000 polarimeter in CHCl_3_. Infrared (IR) spectra (KBr) were recorded on a Nicolet Avatar 320 FT-IR spectrophotometer. Nuclear magnetic resonance (NMR) spectra, including correlation spectroscopy (COSY), nuclear Overhauser effect spectroscopy (NOESY), heteronuclear multiple-bond correlation (HMBC), and heteronuclear multiple quantum coherence (HMQC) experiments, were acquired using a Varian Inova 500 spectrometer operating at 500 MHz (^1^H) and 125 MHz (^13^C), respectively, with chemical shifts given in ppm (δ) using CDCl_3_ as solvent. Chemical shifts were referenced to the residual solvent peaks (δ_H_ 7.24 and δ_C_ 77.0). Mass spectra (EIMS and HREIMS) were recorded on a Finnigan LCQ and JEOL Finnigan MAT 95S Mass Spectrometer, respectively. Column chromatography was performed using silica gel (70-230 mesh, Merck, Darmstadt, Germany) and Sephadex^TM^ LH-20 (Amersham Biosciences, Uppsala, Sweden). Preparative HPLC was conducted using a L-2130 pump (Hitachi, Tokyo, Japan) anda LiChrosorb Si-60 column (Merck).

### 3.2. Plant Material

The aerial parts of *Hypericum sampsonii* Hance were collected from Chia-Yi county in June 2007. The plant was identified by Mr. Jun-Chih Ou, former associate research fellow of National Research Institute of Chinese Medicine, and compared with a voucher specimen which was deposited in the Herbarium of the Institute of Ecology and Evolutionary Biology, National Taiwan University, Taipei, Taiwan (No.077152).

### 3.3. Extraction and Isolation

The dried aerial parts of *H. sampsonii* (12.0 kg) were extracted overnight with 95% ethanol (EtOH) at 60 °C three times (80 L each). The EtOH extracts were concentrated under reduced pressure, and the residue (215 g) was partitioned successively with ethyl acetate (EtOAc) and *n*-butanol (BuOH), respectively. The EtOAc fraction (103 g) was subjected to silica gel column chromatography (8 × 80 cm) and eluted with a EtOAc/hexane gradient. Fractions of 10%–15% EtOAc eluate were collected and rechromatographed over silica gel and RP-18 (MeOH) columns in combination with silica-gel preparative HPLC (15% or 20% EtOAc/Hex) to give hypersampsone R (**1**) (15 mg), hypersampsone S (**2**) (12 mg), and hyperibone K (**3**) (25 mg).

*Hypersamsone* *R* (**1**). Colorless amorphous powder. ${\left[\alpha \right]}_{D}^{25}$: +160 (*c* 0.1, CHCl_3_). IR (KBr): υ_max_ = 3443, 2965, 2928, 1709, 1677, 1598, 1257, 1115, and 747 cm^−1^. ^1^H- and ^1^^3^C-NMR spectroscopic data, see Table 1. Key COSY correlations: H-6/H-7; H-6/H-24; H-7/H-8; H-8/H-12; H-12/H-13; H-24/H-25; H-29/H-30. Key NOESY correlations: H-7/H-10; H-7/H-13; H-7/H-24; H-11/H-12; H-11/H-30; H-12/H-19. Key HMBC correlations: H-6/C-1, -7, -24; H-10/C-2, -8, -9; H-12/C-3, -4, -7, -8, -13, -14, -17; H-13/C-15, -16; H-19/C-17, -18, -20, -21; H-24/C-6, -7, -25, -26; H-29/C-2, -3, -9, -30, -31; H-32 (H-33)/C-30, -31. EI-MS: *m*/*z* (%) = 474 [M]^+^ (8), 446 (15), 128 (23), 105 (100). HR-EI-MS: *m*/*z* = 474.3128 [M]^+^ (calcd for C_32_H_42_O_3_: 474.3134).

*Hypersamsone* *S* (**2**). Colorless amorphous powder. ${\left[\alpha \right]}_{D}^{25}$: +33 (*c* 0.3, CHCl_3_). IR (KBr): υ_max_ 2969, 2927, 1718, 1703, 1441, 1136, 1067, and 826 cm^−1^. ^1^H- and ^1^^3^C-NMR spectroscopic data, see Table 1. Key COSY correlations: H-8/H-7, -12; H-13/H-4, -12, -14; H-24/H-23, -25; H-30/H-31, -32. Key NOESY correlations: H-4/H-13; H-7/H-10, H-14, H-19; H-8/H-7; H-11/H-13, -32; H-15/H-14, -17, -18; H-25/H-23, H-24, -27, -28; H-30/H-31, -32. Key HMBC correlations: H-4/C-2, -3, -6, -12, -14; H-7/C-1, -5, -6, -8, -9, -12; H-12/C-4, -7, -8; H-13/C-4, -5, -12, -14; H-14/ C-4, -12, -13, -15, -16; H-15/C-13, -14, -16, -17, -18; H-17/C-15, -16, -18; H-19/C-1, -5, -6, -7, -20, -21; H-31/C-29, -30, -32. EI-MS: *m*/*z* (%) = 494 [M]^+^ (10), 466 (16), 397 (25), 355 (35), 189 (38), 135 (45), 109 (52), 91 (55), 71 (100). HR-EI-MS: *m*/*z* = 494.3396 [M]^+^ (calcd for C_3__2_H_46_O_4_: 494.3394).

## 4. Conclusions

Three compounds, including two new compounds **1** and **2**, were isolated from the aerial parts of *H. sampsonii*. The structures of these compounds were established on the basis of spectroscopic data. The discovery of new compounds from the genus *Hypericum* may not only provide more spectroscopic data about these isolates, but may also contribute to enhancing our understanding of the taxonomy and evolution of the genus *Hypericum*.
